# Health Literacy and Environmental Risks Focusing Air Pollution: Results from a Cross-Sectional Study in Germany

**DOI:** 10.3390/ijerph21030366

**Published:** 2024-03-19

**Authors:** Elisabeth Pfleger, Hans Drexler, Regina Lutz

**Affiliations:** 1 Institute and Outpatient Clinic of Occupational, Social and Environmental Medicine, Friedrich-Alexander-Universität Erlangen-Nürnberg (FAU), Henkestrasse 9-11, 91054 Erlangen, Germany; hans.drexler@fau.de; 2 FOM Hochschule für Oekonomie & Management, Leimkugelstraße 6, 45141 Essen, Germany; regina.lutz@fom-net.de

**Keywords:** health literacy, environment, public health, air pollution, particulate matter

## Abstract

(1) Background: Environmental risks such as air pollutants pose a threat to human health and must be communicated to the affected population to create awareness, such as via health literacy (HL); (2) Methods: We analyzed HL in the context of environmental health risks, including sources of information and prior knowledge, in a sample from the German general population using Kendall’s rank correlations, regression analyses, and explorative parallel mediation analysis; (3) Results: The survey included 412 German participants aged between 18 and 77. HL was found to be problematic to inadequate. The internet, family and friends, and newspapers were the most frequently cited sources of information. Mobile apps were mostly unknown but were requested by sample subjects. Although subjects expressed environmental concerns and exhibited rather good levels of knowledge, the majority perceived no risk to human health and rated air quality quite positively. Knowledge on particulate matter, the term “ultrafine particles”, and protective measures was found to be rather low. HL was associated with the use of newspapers and commercials as sources of information. The relationship between age and HL is fully mediated by the use of newspapers and information from TV commercials; (4) Conclusions: HL should be promoted by raising awareness of the health effects of environmental pollutants. In particular, the information channels preferred by the affected population should be used and further information opportunities such as apps should be publicized, e.g., through campaigns. An improved HL can assist policy makers in creating a healthier environment by empowering individuals to become more environmentally aware and protect their own health. This, in turn, has the potential to reduce health-related costs.

## 1. Introduction

An essential element for healthy living is an environment which promotes good health. The environment is, however, burdened by many pollutants which contribute to environmental degradation and affect human health and well-being [1,2,3]. Accordingly, air pollution is now considered the greatest environmental threat to human health in violation of basic rights to health and life. As a result, the World Health Organization (WHO) recently published new global air quality guidelines, recommending maximum air quality levels for six pollutants, for which significant evidence on health effects from exposure is available [4]: particulate matter (PM_10_, PM_2.5_), ozone (O_3_), nitrogen dioxide (NO_2_), sulfur dioxide (SO_2_), carbon monoxide (CO), and lead (Pb) [5].

Among these air pollutants, PM in particular poses a particular health risk as it is composed of particles of different sizes. The smaller the particles, the deeper they can penetrate the organism and trigger adverse effects. In this context, ultrafine particles (UFP), the smallest components of PM with a size less than 100nm, are also of increasing scientific importance [6]. In addition to particle size, their surface area, sources of pollution, and chemical composition also play an essential role in health effects. The lower the mass of the particles, the larger the reactive surface. This can increase the biological and chemical activity of the particles [7]. Furthermore, PM can originate from various sources. These include natural sources, such as volcanic eruptions or forest fires, as well as anthropogenic sources, such as traffic or agriculture. Accordingly, the chemical composition of the particles can also vary depending on the sources that interact. Dangerous substances, such as heavy metals and carcinogenic polycyclic aromatic hydrocarbons, can accumulate on the surface of the particles. As a result, the adverse health effects are not only determined by the size of the particles, but also by their chemical combustion and source [7,8].

While the health effects of UFP have not yet been conclusively scientifically established, there are extensive data on the health effects of PM, primarily respiratory and cardiovascular diseases [9,10,11,12,13,14]. In addition, exposure to particulate matter increases the risk of inflammatory diseases such as multiple sclerosis [15], which has been found to occur with chronic exposure to PM_2.5_, even in healthy adults, and increases further with long-term exposure [15,16]. Health effects can emerge in the population at any stage of life; this claim is evidenced by premature births or increased blood pressure in newborns when exposed to elevated levels of particulate matter during pregnancy [17,18,19], as well as impaired cognitive health in old age due to exposure to air pollutants [20,21] or an increased risk of Alzheimer’s dementia after chronic exposure to traffic-related air pollutants [22,23]. Bay-UFP, a research network which is funded by the Bavarian State Ministry for the Environment and Consumer Protection, is currently investigating to what extent UFPs contribute to human health [24].

The WHO has established air-quality guidelines as a global target for political actors to improve public health by reducing air pollution [25]. Based on these recommendations, various nations have already enacted legally prescribed limits for many air pollutants that must be met to avoid adverse effects on human health [26]. In the European Union (EU), for example, such air-quality regulations are stipulated in EU Directive 2008/50/EC. Under this directive, member states are not only required to continuously monitor and document air quality but must also make this information publicly available [27]. Targeted measures, such as air quality indices, have been implemented, for example, which aim to make pollution levels and their harmful effects more widely comprehensible [28,29]. The goal is to enable the affected population to inform themselves about the air quality and the level of air pollution. Furthermore, air-quality guidelines also provide the basis for successful risk communication about the effects of air pollution on human health.

Despite legal limits and measures to mitigate air pollution, however, the average life expectancy in Europe continues to shorten by about 2.2 years [30], and an estimated 4.2 million people worldwide die each year as a result of air pollution [31]. According to the WHO, a rise in particulate matter concentration of 10 µg/m^3^ (PM_2.5_) further increases the risk of death by 8% [4].

Risk communication on environmental health risks regarding air pollution is, therefore, of particular importance [4]. However, in this regard, it is not enough to provide information in accordance with regulations; policy makers must also ensure that the population can also find and understand this information and is able to act accordingly as needed. Health literacy (HL) is a suitable measure of this concept and is defined as follows: “Health literacy implies the achievement of a level of knowledge, personal skills and confidence to take action to improve personal and community health by changing personal lifestyles and living conditions” [32]. HL, therefore, includes not only the ability to read and write but also the knowledge, motivation, and skills to form opinions in the areas of healthcare, disease prevention, and the promotion of health, as well as the ability to make decisions to maintain or improve one’s quality of life [33,34,35,36]. What is understood as HL is not rigidly fixed but rather evolves through the influence of personal, situational, and societal factors, as well as through the acquisition of skills and informal learning over the course of a person’s life [33,37].

Research on HL has been extensive and its impact in the context of various health issues has been examined in earlier publications [38,39,40]. In addition, environmental issues with regard to air pollutants and particulate matter have also been examined in the context of HL. A preliminary systematic review of the scientific literature found 24 studies that analyzed the relationship between HL and environmental issues [41]. Half of the studies analyzed HL in the general population, while the other half looked at specific populations such as students, parents, or patients. In terms of the countries where the studies were carried out, only two studies were conducted in Europe, and none in Germany. Of the studies included in the review, only three related to air pollutants, and only one study, by Hou et al. 2021, was conducted specifically on PM. The review also showed that HL was measured differently in each study, so there is no standardized method of measurement [41]. 

To close this research gap and to promote HL with the aim of increasing public awareness of the harmful effects of these environmental hazards and, thus, empower individuals to protect their health in the long term, we conducted a survey of the German adult population. The study aimed to investigate the level of HL of German adults in relation to air pollutants, specifically particulate matter. As Hou et al. (2021) [42] used a questionnaire based on the European Health Literacy Survey Questionnaire (HLS-EU-Q), we opted to use the established HLS-EU-Q16 questionnaire for Germany. The study by Hou et al. 2021 [42] focused mainly on the development of the survey instrument. In our study we additionally analyzed how the affected population receives and uses information on environmental issues, especially PM. To the best of our knowledge, this study is the first to examine the factors that can contribute to the dissemination of information on particulate matter and its adverse health effects to the affected population in Germany. It also provides suggestions for improvement in the case of low HL levels. This information may be particularly relevant for policy makers working to protect public health.

## 2. Materials and Methods

### 2.1. Study Design

For the present study, a Germany-wide online survey was conducted using a random sample. A questionnaire was developed and pretested. The necessary data protection requirements were reviewed by the Data Protection Officer of FAU Erlangen-Nürnberg. Due to the anonymous survey and the composition of the sample, the vote of an ethics committee was not required. For preliminary testing, we published the questionnaire online for a few days and asked 20 people to complete the questionnaire. The questions were discussed and the content validity of questions not adopted from existing questionnaires were reviewed in detail by a panel of experts from the Institute of Occupational, Social, and Environmental Medicine [43]. Recruitment took place via several routes, i.e., personal communication, the Institute’s website, health promotion networks and various newsletters between May and July 2022. Criteria for eligibility required subjects to be German citizens over the age of 18. A total of 482 people were available for data analysis. Since we used numerous methods of recruitment, no conclusive statement can be made about response rates. 

### 2.2. Measures

Our survey used the German versions of various questionnaires, modified questions from German questionnaires, and self-administered items, all of which are described in the following sections.

To assess participants’ health literacy, we used the HLS-EU-Q16 questionnaire by Röthlin et al. [44]. Each question could be answered on a four-point scale (“very easy”, “fairly easy”, “fairly difficult”, and “very difficult”).

To collect data on information sources and their respective use, we slightly modified the question from Capellaro and Sturm [45] to fit our purpose. We asked: “How often do you use the following sources to find out about environmental issues?” and used the following sources of information: “School/university”, “Workplace/colleagues”, “Occupational physician”, “Information brochures”, “Family/friends”, “Family doctor”, “Newspaper”, “Commercials”, “Internet”, and “Apps” with the answer options, “very frequently”, “frequently”, “occasionally”, “rarely”, “very rarely”, and “never”. We modified the question because we were interested in both environmental issues and health concerns and, in our view, the original question was not specific enough and too few possible answers were given. For the media themselves, we removed any which were not relevant to the fields of occupational and environmental medicine.

As we wanted to know more precisely whether our subjects used specific apps relating to air quality, UV radiation, heat, and pollen, we asked the question “Do you know and use apps relating to the environmental impacts listed?”. For each environmental impact, the following answers were possible: “I know it and use it”, “I know it, but do not use it”, “I don’t know it but would like to use it”, and “I don’t know it and do not want to use it”.

We also used the questions “Are there any environmental issues you are concerned about?” (with answer options “yes” and “no”) and “Please use this list to name the environmental issues that you are concerned about” by Zok and Kolpatzik [46]. In the second question, respondents were asked to indicate whether their concerns related to climate change, plastic/microplastics, the increase in waste in the environment, loss of biodiversity, man-made disasters, pollution, noise pollution, problems in cities (pollution, traffic, lack of green spaces), natural disasters or pollution from agriculture. We slightly summarized and modified these answer options so that fewer categories were available to ensure a more economical survey time. With regard to their own health, we asked subjects a question from to Zok and Kolpatzik [46], “How much do you think pollution and environmental pollutants affect your health?” The question could be answered on a five-point scale with the values “very strongly”, “strongly”, “less strongly”, “not at all”, and “I don’t know”.

We formulated the question “How well informed do you feel about the following topics?” with the topics “heat”, “storms and floods”, “UV radiation”, “air pollution”, and “pollen” to be assessed, based on Schmuker et al. [47]. The response categories were “I know everything about it”, “I know a lot about it”, “I know little about it”, and “I know nothing about it”.

We supplemented the survey with questions on the perception of air quality, “How would you describe the air quality in general in your area?” (“very good”, “rather good”, “rather bad”, “very bad”), “How well informed do you feel about particulate matter?” (“very well-informed”, “well-informed”, “poorly informed”, “very poorly informed”), and “Have you heard about the term ‘UFP’ or ‘ultrafine particles’?” (“yes”, “no”), to obtain a general impression of how the respondents assess the air quality and their knowledge about particulate matter and ultrafine particles.

Regarding the protective measures against particulate matter pollution, we asked the self-written question “Are you aware of protective measures in times of increased particulate matter pollution?” with the answer options “yes” and “no”. In addition, we asked the question, “How do you behave in times of increased particulate matter pollution?”, which was slightly modified from Schmuker et al. [47]. The options “I avoid physical activity outdoors, including sport”, “I keep the windows closed”, “reduce the amount of time I spend outdoors”, and “I use medication to treat the symptoms” were adopted from Schmuker et al. [47] as well. We adapted the response categories to “always”, “often”, “sometimes”, “rarely”, and “never”.

Data were collected using the pretested questionnaire designed as described above (see Supplemental Material, S1: Questionnaire).

### 2.3. Analytic Procedure

Data were analyzed using R Studio, based on R (Version 4.1.2). We first checked which respondents had sufficiently answered the questions on health literacy (a sum value was not calculated for cases of more than two missing values) [44]. For the 412 questionnaires which were sufficiently completed, we calculated reliability measures. With a Cronbach’s α = 0.89, the health literacy exhibited good internal consistency. To calculate the health literacy score, the response categories of the 16 items were dichotomized, as recommended by Röthlin et al. [44]. For the responses “very simple” and “simple”, a value of 1 was assigned, and for the responses “rather difficult” and “very difficult”, a value of 0 was assigned, respectively. The health literacy score was then calculated as a sum score, where all ‘1’ values were added together, allowing individuals to score between 0 and 16 points. Individuals who scored 13 or more points on the HLS-EU-Q16 scale were classified as having ‘adequate’ HL. For individuals scoring between 9–12 points, HL was considered as ‘problematic’. Those who scored 8 or fewer points were considered to have ‘inadequate’ HL.

Descriptive statistics (frequencies, range, mean, standard derivation) were reported for sociodemographic data as well as for HL, environmental questions, and questions regarding informational behavior. For hypothesis testing between HL and environmental questions and questions regarding knowledge and informational behavior, we performed Kendall’s rank correlations, linear regression analyses, and explorative parallel mediation analysis with the “PROCESS” function of Andrew F. Hayes [48]. The raw scores of HL were used for the regression analyses.

## 3. Results

### 3.1. Sociodemographic Characteristics of the Study Population

**Figure 1:**
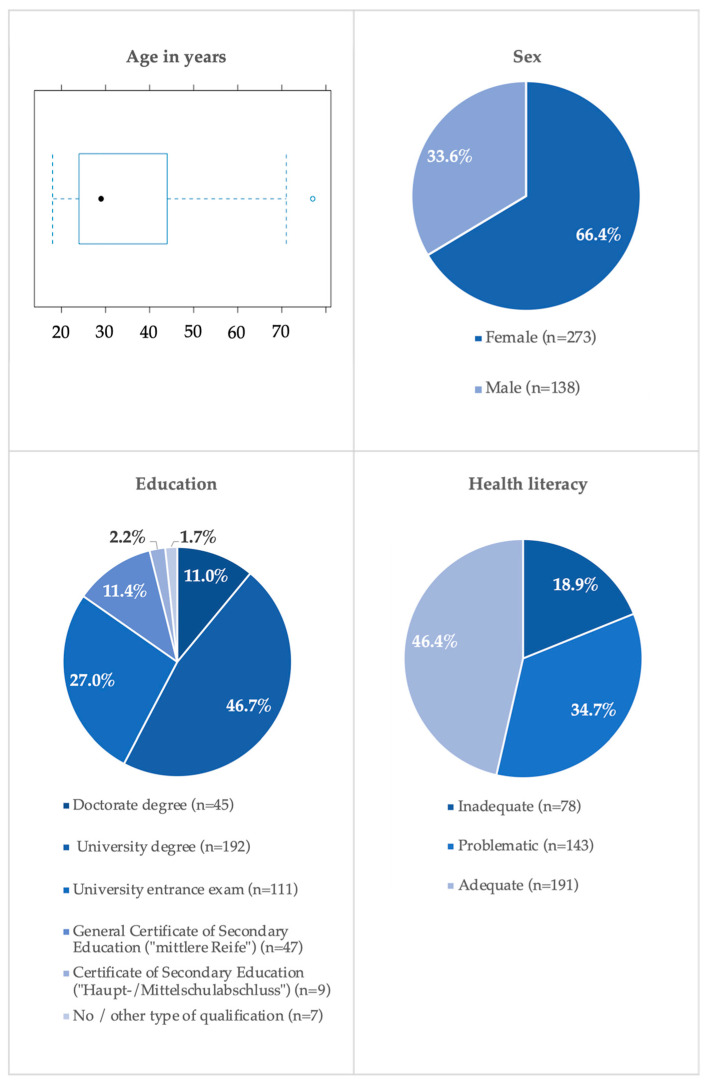
Socio-demographic data (age in years, sex, education) and HL of the study population.

Of all participants, 66.4% (n = 273) of the participants were women and 33.6% (n = 138) were men. Ages ranged from 18 to 77 years with a mean age of 34.5 years (SD = 13.3; n = 405). Most of the respondents reported being highly educated; 27.0% (n = 111) had passed a university entrance exam, 46.7% (n = 192) had completed a university degree, and 11.0% (n = 45) held a doctorate degree. In addition 13.6% (n = 56) hold a secondary school qualification (2.2% (n = 9) have a Certificate of Secondary Education/‘Haupt-/Mittelschulabschluss’ and 11.4% (n = 47) have a General Certificate of Secondary Education/‘mittlere Reife’). Only a small minority had not (yet) completed school or held another type of qualification (1.7%, n = 7). Our study found that the health literacy level was “inadequate” in 18.9% (n = 78), “problematic” in 34.7% (n = 143), and “adequate” in 46.4% (n = 191) of the respondents. Sociodemographic data and HL are shown in Figure 1.

### 3.2. Information Sources, Knowledge, Attitudes, and Behavior Related to Environmental Issues

**Figure 2:**
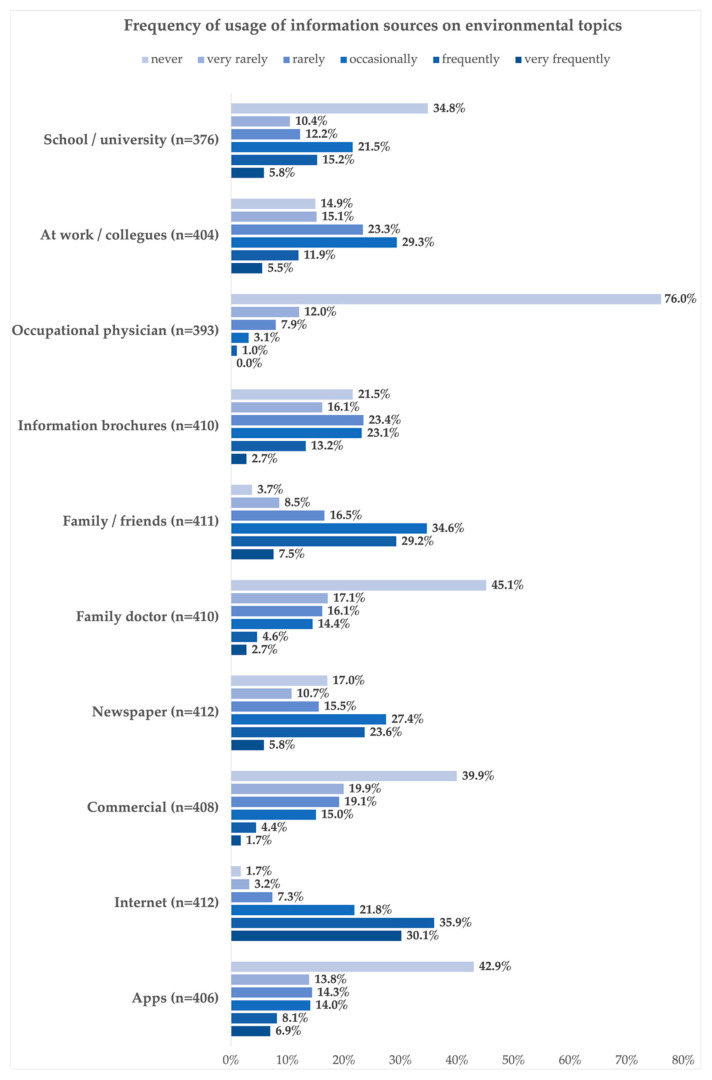
Frequency of use of information sources on environmental topics.

When asked how frequently information on environmental topics was gathered from specified information sources respondents answered that they were most often obtained via the internet (“frequently”: 35.9% (n = 148); “very frequently”: 30.1% (n = 124)), from family or friends (“frequently”: 29.2% (n = 120); “very frequently”: 7.5% (n = 31)), the newspaper (“frequently”: 23.6% (n = 97); “very frequently”: 5.8% (n = 24)) and from their school or university (“frequently”: 15,2% (n = 57); “very frequently”: 5.8% (n = 22)). Information sources that were generally used “very rarely” or even “never” were the occupational physician (“very rarely”: 12,0% (n = 47); “never”: 76.0% (n = 299)) followed by the family doctor (“very rarely”: 17.1% (n = 70); “never”: 45.1% (n = 185)), TV commercials (“very rarely”: 12.0% (n = 81); “never”: 76.0% (n = 163)), and apps (“very rarely”: 13.8% (n = 56); “never”: 42.9% (n = 174)) (see Figure 2).

**Figure 3:**
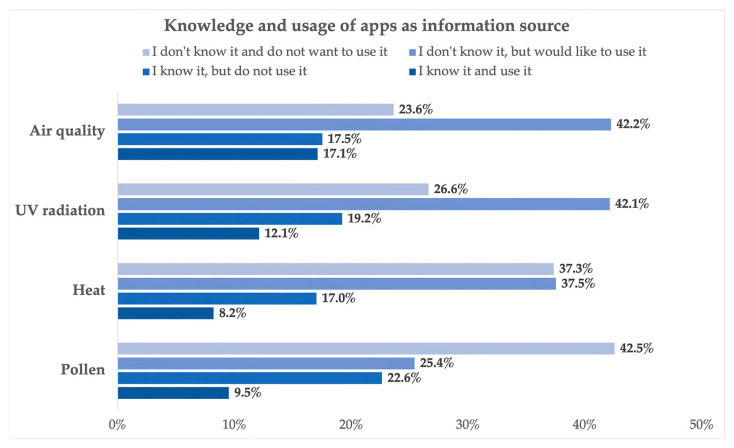
Knowledge and usage of apps as information sources on pollen, heat, UV radiation, and air quality.

An additional question focused on the awareness and use of specific apps for air quality, UV radiation, heat, and pollen, whereby it was established that only a very small proportion of respondents knew of and used apps as a source of information on these environmental risks (air quality: 17.1% (n = 68); UV radiation: 12.1% (n = 49); heat: 8.2% (n = 33); pollen: 9.5% (n = 38)). Regarding the environmental risks of air quality, UV radiation, and heat, most respondents indicated that they were not aware of these apps but would like to use them (air quality: 42.2% (n = 171); UV radiation: 42.1% (n = 171); heat: 37.5% (n = 152)). Apps concerning pollen were not known to most respondents, but were also of no particular interest (42.5%, n = 171) (see Figure 3).

The majority of participants answered “yes” (90.9%, n = 371) to the question of whether they had any concerns about environmental risks. Environmental issues about which most respondents were concerned included climate change (87.1%, n = 323), increase in waste (plastic/microplastic/trash) (83.3%, n = 309), pollution (air/water/soil) (75.7%, n = 281), loss of biodiversity (70.6%, n = 262), anthropogenetic disasters (65.5%, n = 243), and natural disasters (52.8%, n = 196). The topic that caused the least concern was noise (23.7%, n = 88), which was mentioned by less than a quarter of respondents. The impact of environmental pollutants on personal health was assessed as “less strong” by 46.7% (n = 191) of the respondents. In contrast, 36.7% (n = 150) of respondents considered their health to be “strongly” impacted, and 11.2% (n = 46) were reportedly “very strongly” impacted. Moreover, 2.5% (n = 10) assessed their personal health as “not at all” impacted and 2.9% (n = 12) stated that they did not know whether or to what extent their health may be impacted by environmental pollutants.

**Figure 4:**
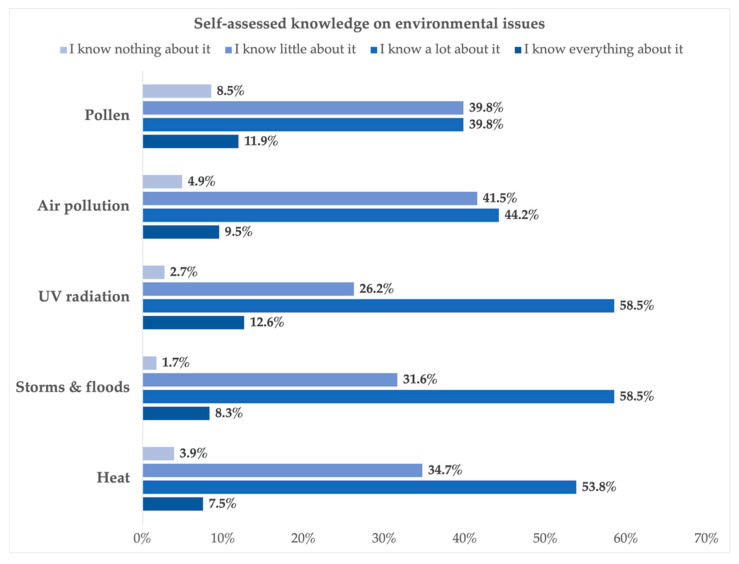
Self-assessed knowledge on pollen, air pollution, UV radiation, storms and floods, and heat.

When asked about their self-assessed knowledge with respect to heat, storms and floods, UV, air pollution, and pollen, most respondents answered that they know a lot about it (heat: 53.8% (n = 222); storms and floods: 58.5% (n = 241); UV: 58.5% (n = 241); air pollution: 44.2% (n = 182); pollen: 39.8% (n = 164)) followed by those who reported to know little about it (heat: 34.7% (n = 143); storms and floods: 31.6% (n = 130); UV radiation: 26.2% (n = 108); air pollution: 41.5% (n = 171); pollen: 39.8% (n = 164)). The statements “I know everything about it” and “I know nothing about it” were only mentioned by a small proportion of the respondents (see Figure 4).

When asked how they perceive air quality in their surroundings, more than two-thirds of respondents answered “rather good” (67.4%, n = 271), followed by “rather bad” (21.4%, n = 86). A smaller proportion rated the air quality as “very good” (9.2%, n = 37) and 2% (n = 8) perceived it as “very bad”.

On the question of how well-informed respondents felt about particulate matter, just under half of respondents answered that they feel “poorly informed” (47.5%, n = 183), followed by “very poorly informed” (18.7%, n = 72). Other respondents reportedly felt “well informed” (27.5%, n = 106) and only 6.3% (n = 24) felt “very well-informed”. The terms “UFP” or “ultrafine particles” in the context of air pollution were known to 43.2% (n = 176) of respondents, while 56.8% (n = 233) stated that they had never heard of these terms before.

On the question of whether protective measures in times of increased particulate matter pollution were known, 66.8% (n = 274) answered with “no”. Among those who stated that they were aware of such protective measures (33.2%, n = 136), it was queried how often specific measures were applied. Avoiding outdoor physical activity, including sports, was implemented by 12.5% (n = 16) “always”, 31.3% (n = 40) “often”, 28.9% (n = 37) “sometimes”, 13.2% (n = 17) “rarely”, and 14.1% (n = 18) “never”. Windows were kept closed by 15.6% (n = 20) of participants “always”, 38.3% (n = 49) “often”, 20.3% (n = 26) “sometimes”, 15.6% (n = 20) “rarely”, and 10.2% (n = 13) “never”. Time spent outdoors was reduced from 10.2% (n = 13) “always”, 22.0% (n = 28) “often”, 33.1% (n = 42) “sometimes”, 21.3% (n = 27) “rarely”, and 13.4% (n = 17) “never”. Medication to treat relevant symptoms was taken only “rarely” (14.0%, n = 18) or “never” (79.0%, n = 102). Only 3.9% (n = 5) stated to use such medications “sometimes” and 2.3% (n = 3) “often”, and 0.8% (n = 1) reported using it “always”.

### 3.3. Association between Health Literacy and Environmental Issues

Self-assessed knowledge of heat, storms and floods, UV radiation, air pollution, and pollen were significantly and positively correlated with the level of HL, showing that the higher the level of HL, the better the self-assessed knowledge of all specified environmental issues. The results of the correlation analyses are shown in Table 1.

With regard to the frequency of executing specific protective measures in the event of increased PM exposure, we found no significant effects in the performed regression analysis. 

In terms of how frequently certain information sources are used (Model 1), the regression model appears significant, and a higher HL value was significantly associated with a more frequent use of newspapers, while a lower HL value was significantly associated with an increased use of TV commercials.

According to Gray et al. [49] and Zhao et al. [50], lower age should be significantly related to a higher HL value. We modified our regression model and exploratively tested whether age and the information sources “newspapers” and “TV commercials” showed a better fit (Model 2). Surprisingly, the significant effects of the given information sources disappeared completely when we added the age predictor (see Table 2).

Therefore, we assumed that the information sources could possibly be mediator variables and investigated this hypothesis with a parallel mediation analysis using PROCESS. The outcome variable for analysis was HL, the predictor variable was age, and the mediator variables were “use of newspapers” and “use of TV commercials”. Results indicated that age is indirectly related to HL via its relationship with the frequency of use of newspapers and of TV commercials. 

**Figure 5:**
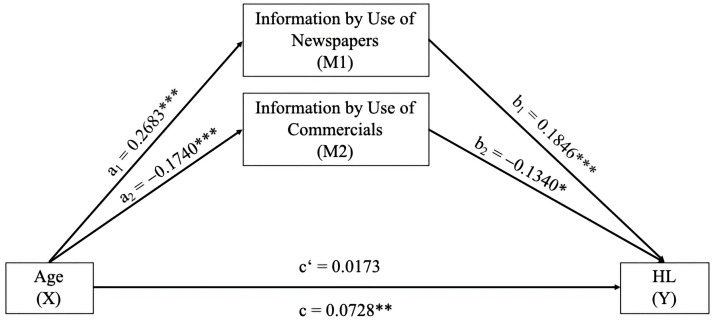
The mediating effect of information sources in the relationship between age and HL. Notes: * *p* < 0.05, ** *p* < 0.01, *** *p* < 0.001. All presented effects are totally standardized; a_n_ is the effect of age on information sources; b_n_ is the effect of information sources on HL; c’ is the direct effect of age on HL; c is the total effect of age on HL.

First, as can be seen in Table 3, higher age is related to more frequent use of newspapers (a_1_), and more frequent use of newspapers was subsequently related to a higher HL value (b_1_). Second, higher age is related to lower use of TV commercials (a_2_), and lower use of commercials was subsequently related to a lower HL value (b_2_). A 95% bias-corrected confidence interval based on 10,000 bootstrap samples indicated that the indirect effect through newspapers as an information source (a_1_b_1_), holding the second mediator constant, was entirely above zero. In addition, the indirect effect by the use of commercials (a_2_b_2_), holding the first mediator constant, was also entirely above zero. While the total effect of age on HL was significant (c), the direct effect was not (c’). We found that the relationship between age and HL is fully mediated by the use of newspapers and information by commercials (IE overall) (see Figure 5).

## 4. Discussion

### 4.1. Discussion of the Results

The relationship between HL and environmental issues has already been investigated in numerous studies throughout the world [41]. This study is the first to provide data on this relationship in Germany.

Previous studies have shown that a higher level of HL is often associated with a higher level of education. Although our sample consists largely of highly educated participants, the majority of respondents were found to have an inadequate or problematic level of HL. This result is, therefore, inconsistent with other studies that have also analyzed HL in highly educated participants, e.g., students, and found an adequate to high level of HL [49,51,52,53]. In contrast, studies analyzing HL in the general population, within which all educational levels were represented, found an inadequate or problematic level of HL [42,49,54]. The fact that our population consists of generally rather highly educated participants as well as people with a medium or lower level of education could explain the lower level of HL in our sample.

Regarding information sources, it was found that the most frequently used sources of environmental information are the internet, family and friends, and the newspaper. These results are in line with the findings of a study analyzing the usage frequency of sources for health information [45]. This result is promising as it means that both environmental and health-related topics can be disseminated together via one medium, allowing relevant information to reach a large part of the population in a compact form. 

While doctors are also a frequently used information source for health issues [45], both the occupational physicians and the family doctors are only very rarely or even never used as sources of information on environmental issues by the majority of respondents in our sample. This finding indicates that environmental pollutants are seen less as a risk to human health and much more as a threat to nature, such that the population may not recognize the need to discuss these risks with a medical professional [55]. Both awareness of the health risks posed by environmental pollutants and the role of doctors as points of contact on this topic should, therefore, be strengthened [56]. 

Another finding that is consistent with the results of Capellaro and Sturm [45] is that TV commercials and apps are rarely used for informational purposes for either health topics or for environmental issues. The low usage of TV commercials could be due to the fact that linear television is being used less and less, especially within the younger population [57], and that ad blockers are active on many devices, such that advertisements no longer appear at all [58]. 

Another interesting result is the low usage of apps, concluded from our participants’ knowledge and usage of apps as a source of information. Our data showed that the majority of respondents were not aware of apps on heat, UV radiation, and air quality, but would like to use them. However, as the number of smartphone users increases every year [59] and as there are already a large number of apps with relevant information, such as the Federal Environment Agency’s air quality app or many already pre-installed weather apps which come pre-installed on smartphones, there seems to be a problem in terms of the awareness of certain apps or a problem in finding them [60]. As these smartphone technologies offer significant potential to improve public health by enabling the provision of customized, easily accessible health information, their existence should be increasingly communicated to the affected population and the awareness of these apps should be increased [61]. Apps with information on pollen were not known by the majority of respondents but were not considered interesting either. This finding could be due to the fact that pollen is primarily relevant for allergy sufferers and does not affect the entire population in the same way as particulate matter or UV [62].

Concern about the environment has been growing among the German population for several years now as environmental issues have become increasingly noticeable in Germany [63]. Our results also support this fact, with the majority of participants stating that they have environmental concerns. The environmental concerns indicated in our study are quite consistent with the data from a survey from 2020 which was also conducted within the German general population in [46]. Compared to the 2020 study, our 2022 survey found a slight increase in concern about climate change (2020: 78.8% vs. 2022: 87.1%), plastic/microplastic/trash (2020: 77.0% vs. 2022: 83.3%), pollution (2020: water 79.9%/air 56.1%/soil 51.6% vs. 2022: water/air/soil 75.7%), and natural disasters (2020: 52.4% vs. 2022: 52.8%). Compared to the study from 2020, our data found a slight decrease in concern about the loss of biodiversity (2020: 73.8% vs. 2022: 70.6%), anthropogenetic disasters (2020: 66.2% vs. 2022: 65.5%), and noise (2020: 30.0% vs. 2022: 23.7%). The further increase in concern reported in our study—regarding, for example, climate change or natural disasters—could stem from the fact that extreme weather events caused by climate change, such as extreme heat [64] or flooding caused by heavy rainfall, as was seen in the Ahr valley in 2021, have increased in Germany [65], and the population has become more aware of this issue as a result. This finding is also consistent with a 2018 study in Potsdam, Germany in which climate change was likewise perceived as a greater concern than air pollution [66]. 

Compared to data from 2020, awareness that environmental pollutants have a negative impact on health appears to have improved somewhat, as more people in our study state that environmental pollutants have a “very strong” impact on health (2020: 4.8% vs. 2022: 11.2%) and fewer state “not at all” (2020: 8.0% vs. 2022: 2.5%) or “I don’t know” (2020: 7.0% vs. 2022: 2.9%) than in 2020 [46]. Another finding in support of this claim is that most respondents reported to know a lot about environmental topics like heat, storms and floods, UV radiation, air pollution, and pollen. While these results initially appear promising, as an increased awareness of environmental pollutants could increase the focus on protecting one’s own health from these stressors, a further result clouds this hope somewhat, as more than two-thirds of respondents assessed the air in their environment as “rather good”.

Although air pollution in Europe and, therefore, in Germany has decreased in recent decades, there are still numerous limit values that are repeatedly exceeded in this country and, therefore, pose a risk to public health [67,68]. 

Considering environmental risks a threat to health, while air quality is predominantly rated as “rather good”, suggests that the health risks from environmental pollutants are less strongly associated with pollutants in the air, and that other environmental risks, such as climate change or water pollution, are seen as more threatening [46]. Another explanation for this discrepancy could be that, although air pollution is perceived as an environmental risk, it is considered less serious in Germany compared to other countries, such as in Asia [69]. This distorted assessment of air quality is further underlined by the fact that around two-thirds of the respondents reported feeling “rather poorly” or even “very poorly” informed about PM, one of the most prominent air pollutants. The respondents’ low level of knowledge about particulate matter was also reflected in the fact that only around 50% had ever heard the term “ultrafine particles” or “UFP”, and only a third stated that they were aware of protective measures in times of increased PM levels, which, in turn, only a small number of participants have “always” or at least “frequently” implemented. A survey from 2021 similarly found a low level of implementation of protective measures, whereby the most commonly implemented measures also included reducing outdoor activities (29% always/often) or closing windows (28% always/often), and only a few reduced their time spent outdoors (18% always/often) or used medication (6% always/often) [47].

Significant effects emerged when examining health literacy in the context of environmental issues with respect to sources of information and prior knowledge.

Regarding the frequency of use of information sources for environmental information, a significant effect on HL was found with respect to newspaper and TV commercials, as has been found in previous studies, stating that a lower HL is related to a lower use of newspapers [70] but a higher use of TV commercials [71] regarding health information. The fact that the usage of TV commercials is associated with lower HL could be due to its role as a biased source providing misleading information to influence recipients in the service of specific goals, as commercial goals may override informational goals [72,73]. In order to increase HL in this target group, greater emphasis could be placed on providing the medium of advertising with qualified content, for example, in the form of campaigns that refer to relevant further information, as is already being implemented with regard to vaccination campaigns like “Germany is looking for the vaccination certificate” [74].

Contrary to the relationship between age and HL described in the existing literature [49,50], our data exhibited a positive relationship between age and the HL level. This effect was, however, removed by full mediation with the use of information sources (newspapers, commercials). As our survey comprises a non-representative sample with a rather young average age of 34.5 years, this effect should be further investigated in a more representative sample. 

In addition, our results show that a higher HL has a positive relationship with self-assessed knowledge of environmental issues like heat, UV radiation, storms and floods, air pollution, and pollen. This finding very well supports those of previous studies, which also found that people with higher HL had more knowledge about the risks of environmental pollutants than those with lower HL [75]. These results are promising in that they show that public knowledge can be improved by investing in HL, which can, in turn, bring more focus to personal health risks related to environmental issues.

### 4.2. Limitations

There are certain limitations to our study. First, our data are not representative of the German population and only provide an initial insight into a small section of the population, as we used a random sample. For a representative picture and more reliable data, studies should be carried out on additional, larger samples. Second, genders were not equally distributed within our population, with considerably more women taking part in the survey than men. In addition, the participants in our study were rather young, meaning that the conclusiveness of the results may be limited with respect to the older population. Our sample also included many highly educated participants, which could also distort the results with respect to people with lower levels of formal education. Since we conducted a cross-sectional study, we only measured health literacy at one point in time. It may, therefore, be more informative to examine this measure on a longitudinal basis to investigate changes over time as well as reactions to changing circumstances. The study variables were measured based on self-reporting, which is commonly known to be biased. As a result, social desirability cannot be completely excluded. To avoid this problem, participants were assured of strict anonymity [76]. Regarding the questionnaire, we used some self-written questions. Because most of our self-written questions were used to assess the use of different information sources (which would rather represent a manifest variable), we could not validate these questions in a manner typical for latent variables. As such, it may be advisable to redesign the questionnaire, for example, to include the use of other latent constructs (e.g., environmental health literacy). Since the study is largely based on responses collected via a distributed questionnaire, an additional question should be included for subsequent research to further confirm the validity of the responses and ensure the integrity of the data collection process.

## 5. Conclusions

Although numerous measures have already been taken to create a healthy environment in Germany, environmental risks such as climate change and air pollutants remain serious issues whose effects are highly relevant to public health [77]. Health literacy plays an essential role in comprehensive educational work and public awareness, as it enables individuals to make decisions in their private lives that positively impact their own health [37]. Our initial data show that it is essential to increase this awareness in the German general population. Since the internet, family and friends, and newspapers, were found to be the preferred sources of information on environmental issues, there is a possibility of increasingly providing these sources with health-related information on environmental issues, whereby particular care must be taken to ensure that this information is easy to find and understand. As social interactions with peers are also frequently cited as a source of such information, discourse should be further stimulated in this context by providing high-quality information so that this information can be passed on among individuals. However, it is not enough to simply provide information, e.g., via apps or an air quality index, as the affected population may not be sufficiently aware of them. There is rather a need to actively impart and increase the knowledge of information sources, and greater attention must be paid to ensuring that the population is aware of opportunities to obtain such information themselves. Furthermore, HL initiatives must not rely solely on people searching for information on their own initiative, but it must be focused on the active transfer of knowledge, as training courses, for example, have proven to be very effective measures for increasing HL, as has been proven in the context of environmental risks in other countries [41]. For this reason, it is advisable for the German population to focus on the possibility of promoting HL, such as by offering relevant instruction in schools or community centers in order to make the population more aware of these risks and to promote individual action, including taking protective measures as needed. Improved health literacy can assist policy makers in creating a healthy living environment. By improving health literacy, individuals can make informed decisions about their health and take action to improve their personal and community health. This includes changing personal lifestyles and living conditions to reduce exposure to particulate matter pollution, leading to a healthier life and potentially also reducing health-related costs [78].

Further research is necessary to confirm and strengthen the findings of this study, including those which concern environmental health literacy. 

## Figures and Tables

**Table 1:** Statistical results of the correlation analyses (Kendall’s rank correlations, τ) between HL and self-assessed knowledge of environmental risks (heat, storms and floods, UV radiation, air pollution, and pollen).

Variables		HL
HL		1
Self-assessed knowledge on:	heat	0.18 ***
	storms and floods	0.22 ***
	UV radiation	0.23 ***
	air pollution	0.19 ***
	pollen	0.19 ***

Note: *** indicates *p* < 0.001.

**Table 2:** Regression analysis of the relationship between HL and the frequency of use of different information sources (Model 1) and age (Model 2).

	Model 1					Model 2				
	Est.	SE	*t*-Value	*p*-Value	β	Est.	SE	*t*-Value	*p*-Value	β
Intercept	9.73	0.95	10.187	<0.001	-	8.79	1.12	7.85	<0.001	-
School/university	0.01	0.11	0.08	0.94	0.00	0.09	0.12	0.76	0.45	-
At work/collegues	0.21	0.14	1.45	0.14	0.08	0.18	0.14	1.26	0.21	-
Occupational physician	−0.41	0.27	−1.55	0.12	−0.10	−0.39	0.27	−1.44	0.15	-
Information brochures	0.09	0.16	0.58	0.56	0.04	0.07	0.16	0.46	0.64	-
Family/friends	−0.07	0.17	−0.41	0.68	−0.02	−0.04	0.17	−0.21	0.83	-
Family doctor	0.15	0.15	1.00	0.32	0.06	0.09	0.16	0.59	0.56	-
Newspaper	0.35	0.15	2.37	0.02 *	0.15	0.29	0.15	1.89	0.06	-
Commercial	−0.32	0.15	−2.15	0.03 *	−0.12	−0.27	0.16	−1.71	0.09	-
Internet	0.19	0.19	1.06	0.29	0.07	0.20	0.19	1.10	0.27	-
Apps	0.02	0.12	0.19	0.85	0.01	0.03	0.12	0.26	0.79	-
Age						0.02	0.02	1.41	0.16	-

Note: * indicates *p* < 0.05; Est.: estimates; SE: standard error; Model 1—F(10/346) = 2.25, *p* < 0.05, R^2^adj. = 0.02; Model 2—F(11/241) = 2.22, *p* < 0.05, R^2^adj. = 0.04.

**Table 3:** Results of the parallel mediation analysis of direct, indirect, and total effects of age on HL mediated by the frequency of use of “newspapers” and “commercials” as information sources.

Pathway		Effect	Coef.	95% CI
a_1_	Age → Newspaper	direct	0.2683 ***	0.0199; 0.0415
a_2_	Age → Commercial	direct	−0.1740 ***	−0.0272; −0.0077
b_1_	Newspaper → HL	direct	0.1846 ***	0.1807; 0.6587
b_2_	Commercial → HL	direct	−0.1340 *	−0.6128; −0.0807
a_1_b_1_	Age → Newspaper → HL	indirect	0.0496	0.0198; 0.0845
a_2_b_2_	Age → Commercial → HL	indirect	0.0233	0.0043; 0.0482
c’	Age → HL	direct	0.0173	−0.0095; 0.0441
c	Age →→ HL	IE overall	0.0728 **	0.0331; 0.1162

Note: * indicates *p* < 0.05; ** indicates *p* < 0.01; *** indicates *p* < 0.001.

## Data Availability

The datasets analyzed as part of the current study are not publicly available due to German national data protection regulations but can be made available by the corresponding author upon reasonable request.

## Supplementary Materials

The following supporting information can be downloaded at: https://www.mdpi.com/article/10.3390/ijerph21030366/s1, S1: Questionnaire.
