# Supplementary material for: Health Literacy and Environmental Risks Focusing Air Pollution: Results from a Cross-Sectional Study in Germany
Source: Int J Environ Res Public Health. 2024 Mar 19;21(3):366. doi: 10.3390/ijerph21030366 (PMC10970231; doi:10.3390/ijerph21030366)
Supplement: Supplementary file 1 [file ijerph-21-00366-s001.zip › ijerph-2856856-supplementary.pdf]

Questionnaire

SOCIODEMOGRAPHIE	
**What is your sex?**	o Female<br>o Male<br>o Divers<br><br>o No answer
**What is your age?**	____ years<br><br>o No answer
**What is your highest educational level?**	o No degree<br>o Pupil<br>o Certificate of Secondary Education<br>o General Certificate of Secondary Education<br>o University entrance exam<br>o University degree (bachelor, master, diploma)<br>o Doctoral degree<br>o Other<br><br>o No answer

HEALTH LITERACY	
**On a scale from very easy to very difficult, how easy would you say it is to....**	
**… find information on treatments of illness that concern you?**	o Very easy<br>o Fairly easy<br>o Fairly difficult<br>o Very difficult<br><br>o No answer
**… find out where to get professional help when you are ill?**	o Very easy<br>o Fairly easy<br>o Fairly difficult<br>Very difficult<br><br>o No answer
**… understand what your doctor says to you?**	o Very easy<br>o Fairly easy<br>o Fairly difficult<br>o Very difficult<br><br>o No answer
**… understand your doctor's or pharmacist's instruction on how to take a prescribed medicine?**	o Very easy<br>o Fairly easy<br>o Fairly difficult<br>o Very difficult<br><br>o No answer

**… judge when you may need to get a second opinion from another doctor?**	o Very easy<br>o Fairly easy<br>o Fairly difficult<br>o Very difficult<br><br>o No answer
**… use information the doctor gives you to make decisions about your illness?**	o Very easy<br>o Fairly easy<br>o Fairly difficult<br>o Very difficult<br><br>o No answer
**… follow the instructions from your doctor or pharmacist?**	o Very easy<br>o Fairly easy<br>o Fairly difficult<br>o Very difficult<br><br>o No answer
**… find information on how to manage mental health problems like stress or depression?**	o Very easy<br>o Fairly easy<br>o Fairly difficult<br>o Very difficult<br><br>o No answer
**… understand health warnings about behavior such as smoking, low physical activity and drinking too much?**	o Very easy<br>o Fairly easy<br>o Fairly difficult<br>o Very difficult<br><br>o No answer
**… understand why you need health screening?**	o Very easy<br>o Fairly easy<br>o Fairly difficult<br>o Very difficult<br><br>o No answer
**… judge if the information on health risks in the media is reliable?**	o Very easy<br>o Fairly easy<br>o Fairly difficult<br>o Very difficult<br><br>o No answer
**… decide how you can protect yourself from illness based on information in the media? (e.g. newspaper, brochures, internet, or other media)**	o Very easy<br>o Fairly easy<br>o Fairly difficult<br>o Very difficult<br><br>o No answer

**… find out about activities that are good for your mental well-being? (e.g. drinking and eating habits, exercise, etc.)**	o Very easy<br>o Fairly easy<br>o Fairly difficult<br>o Very difficult<br><br>o No answer
**… understand advice from family members or friends?**	o Very easy<br>o Fairly easy<br>o Fairly difficult<br>o Very difficult<br><br>o No answer
**… understand information in the media on how to get healthier?**	o Very easy<br>o Fairly easy<br>o Fairly difficult<br>o Very difficult<br><br>o No answer
**… judge which everyday behavior is related to your health?**	o Very easy<br>o Fairly easy<br>o Fairly difficult<br>o Very difficult<br><br>o No answer

INFORMATION SOURCES ON ENVIRONMENTAL TOPICS

How often do you use the following sources to find out about environmental issues?

**- School/university**	o Very frequently<br>o Frequently<br>o Occasionally<br>o Rarely<br>o Very rarely<br>o Never<br><br>o No answer
**- Workplace/colleagues**	o Very frequently<br>o Frequently<br>o Occasionally<br>o Rarely<br>o Very rarely<br>o Never<br><br>o No answer
**- Occupational physician**	o Very frequently<br>o Frequently<br>o Occasionally<br>o Rarely<br>o Very rarely<br>o Never<br><br>o No answer

**- Information brochures**	o Very frequently<br>o Frequently<br>o Occasionally<br>o Rarely<br>o Very rarely<br>o Never<br><br>o No answer
**- Family/friends**	o Very frequently<br>o Frequently<br>o Occasionally<br>o Rarely<br>o Very rarely<br>o Never<br><br>o No answer
**- Family doctor**	o Very frequently<br>o Frequently<br>o Occasionally<br>o Rarely<br>o Very rarely<br>o Never<br><br>o No answer
**- Newspaper**	o Very frequently<br>o Frequently<br>o Occasionally<br>o Rarely<br>o Very rarely<br>o Never<br><br>o No answer
**- Commercials**	o Very frequently<br>o Frequently<br>o Occasionally<br>o Rarely<br>o Very rarely<br>o Never<br><br>o No answer
**- Internet**	o Very frequently<br>o Frequently<br>o Occasionally<br>o Rarely<br>o Very rarely<br>o Never<br><br>o No answer

**- Apps**	o Very frequently<br>o Frequently<br>o Occasionally<br>o Rarely<br>o Very rarely<br>o Never<br><br>o No answer
**Do you know and use apps relating to the environmental impacts listed?**	
**- Pollen**	o I know it and use it<br>o I know it, but I don’t use it<br>o I don’t know it, but would like to use it<br>o I don’t know it and do not want to use it<br><br>o No answer
**- Heat**	o I know it and use it<br>o I know it, but I don’t use it<br>o I don’t know it, but would like to use it<br>o I don’t know it and do not want to use it<br><br>o No answer
**- UV radiation**	o I know it and use it<br>o I know it, but I don’t use it<br>o I don’t know it, but would like to use it<br>o I don’t know it and do not want to use it<br><br>o No answer
**- Air quality**	o I know it and use it<br>o I know it, but I don’t use it<br>o I don’t know it, but would like to use it<br>o I don’t know it and do not want to use it<br><br>o No answer
**ENVIRONMENTAL QUESTIONS**	
**Are there any environmental issues you are concerned about?**	o Yes<br>o No<br><br>o No answer
**Please use this list to name the environmental issues that you are concerned about *(multiple choice)*:**	o Climate change<br>o Increase in waste in the environment (plastic/microplastic/trash)<br>o Loss of biodiversity<br>o Anthropogenetic disasters<br>o Pollution (air, water, soil)<br>o Noise pollution<br>o Natural disasters<br><br>o No answer

**How much do you think pollution and environmental pollutants affect your health?**	o Very strong<br>o Strong<br>o Less strong<br>o Not at all<br>o I don't know<br><br>o No answer
**How well informed do you feel about the following topics?**	
**- Heat**	o I know everything about it<br>o I know a lot about it<br>o I know little about it<br>o I know nothing about it<br><br>o No answer
**- Storms and floods**	o I know everything about it<br>o I know a lot about it<br>o I know little about it<br>o I know nothing about it<br><br>o No answer
**- UV radiation**	o I know everything about it<br>o I know a lot about it<br>o I know little about it<br>o I know nothing about it<br><br>o No answer
**- Air pollution**	o I know everything about it<br>o I know a lot about it<br>o I know little about it<br>o I know nothing about it<br><br>o No answer
**QUESTIONS ON AIR POLLUTION & PARTICULATE MATTER**	
**How would you describe the air quality in general in your area?**	o Very good<br>o Rather good<br>o Rather bad<br>o Very bad<br><br>o No answer
**How well informed do you feel about particulate matter**	o Very well informed<br>o Well informed<br>o Poorly informed<br>o Very poorly informed<br><br>o No answer

**Have you heard about the term "UFP" or "ultrafine particles"?**	○ **Yes**<br>○ **No**<br><br>○ **No answer**
**Are you aware of protective measures in times of increased particulate matter pollution?**	○ Yes<br>○ No<br><br>○ No answer
**How do you behave in times of increased particulate matter pollution?**	○ Always<br>○ Often<br>○ Sometimes<br>○ Rarely<br>○ Never<br><br>○ No answer
**- I avoid physical activity outdoors, including sport**	○ Always<br>○ Often<br>○ Sometimes<br>○ Rarely<br>○ Never<br><br>○ No answer
**- I keep the windows closed**	○ Always<br>○ Often<br>○ Sometimes<br>○ Rarely<br>○ Never<br><br>○ No answer
**- I reduce the amount of time I spend outdoors**	○ Always<br>○ Often<br>○ Sometimes<br>○ Rarely<br>○ Never<br><br>○ No answer
**- I use medication to treat the symptoms**	○ Always<br>○ Often<br>○ Sometimes<br>○ Rarely<br>○ Never<br><br>○ No answer
